# Simultaneous Analysis of a Combination of Anti-Hypertensive Drugs, Fimasartan, Amlodipine, and Hydrochlorothiazide, in Rats Using LC-MS/MS and Subsequent Application to Pharmacokinetic Drug Interaction with Red Ginseng Extract

**DOI:** 10.3390/toxics10100576

**Published:** 2022-09-30

**Authors:** So-Yeon Jeon, Ji-Hyeon Jeon, Jin-Hyang Park, Jihoon Lee, Minyeong Pang, Min-Koo Choi, Im-Sook Song

**Affiliations:** 1 College of Pharmacy, Dankook University, Cheon-an 31116, Korea; 2 BK21 FOUR Community-Based Intelligent Novel Drug Discovery Education Unit, Vessel-Organ Interaction Research Center (VOICE), Research Institute of Pharmaceutical Sciences, College of Pharmacy, Kyungpook National University, Daegu 41566, Korea

**Keywords:** fimasartan, amlodipine, hydrochlorothiazide, red ginseng extract, drug interaction

## Abstract

Fimasartan, amlodipine, and hydrochlorothiazide are commonly used in combination therapies as antihypertensive drugs. This study aimed to develop and validate an analytical method for fimasartan, its active and major metabolite fimasartan-amide, amlodipine, and hydrochlorothiazide in rat plasma using liquid chromatography-tandem mass spectrometry (LC-MS/MS). The standard calibration curves for fimasartan (1–500 ng/mL), its active and major metabolite fimasartan-amide (0.3–100 ng/mL), amlodipine (0.5–200 ng/mL), and hydrochlorothiazide (5–5000 ng/mL) were linear with R^2^ > 0.9964, and the inter- and intra-day accuracy and precision and stability were within the acceptable criteria. Using this validated analytical method, the pharmacokinetic interaction of these triple combination drugs between single administration and concomitant administration of the triple combination was investigated; the results did not reveal a significant difference in any of the pharmacokinetic parameters. Based on these results, we investigated the effects of red ginseng extract (RGE) on the pharmacokinetics of fimasartan, fimasartan-amide, amlodipine, and hydrochlorothiazide after oral administration of the combination in rats. No significant difference was observed in the pharmacokinetic parameters of fimasartan, fimasartan-amide, amlodipine, and hydrochlorothiazide, except for the T_max_ values of amlodipine. The delayed T_max_ value of amlodipine was attributed to its decreased intestinal permeability after repeated RGE treatments. In conclusion, using a combination of antihypertensive drugs and simultaneous analytical methods, we established efficient drug interaction and toxicokinetic studies using a small number of animals.

## 1. Introduction

For the management of hypertension, approximately 70% of patients with hypertension take two or more antihypertensive drugs for effective blood pressure control. Among these combination drugs, calcium channel blockers, angiotensin II receptor antagonists, and thiazide diuretics are the most commonly used [1]. Fimasartan is an angiotensin II receptor antagonist that has been approved in Korea and several Latin American countries for the treatment of mild-to-moderate hypertension. It has often been prescribed in combination with amlodipine and/or hydrochlorothiazide in patients with hypertension, as these combination therapies exhibit additive effects [2,3,4]. In addition, fimasartan-amlodipine combination has led to similar adverse events compared with either monotherapy and resulted in a high compliance rate (>90%). Based on these advantages, fimasartan is increasingly used in multiple combination therapies [4]. The combined use of drugs with different mechanisms of action may provide advantages in terms of efficacy and tolerability; however, it also increases the possibility of drug–drug interactions (DDIs). Therefore, the tolerability and safety of combination drugs were evaluated in the context of their DDI potential. Co-administration of fimasartan and amlodipine did not result in substantial changes in the pharmacokinetics of either drug after multiple oral doses [3]. Similarly, no clinically relevant DDIs have been reported for the combination of fimasartan and hydrochlorothiazide, although fimasartan has a slight potential to increase the urinary excretion of hydrochlorothiazide [5]. A fixed-dose triple combination of fimasartan/amlodipine/hydrochlorothiazide 60/10/25 mg exhibited pharmacokinetic profiles similar to those of the corresponding doses of the three drugs [6]. In addition, a physiologically based pharmacokinetic (PBPK) model for fimasartan, amlodipine, and hydrochlorothiazide was developed by Rhee et al., and they predicted no remarkable DDIs using this PBPK model when fimasartan (120–240 mg) was co-administered with amlodipine (10 mg) and hydrochlorothiazide (25 mg), which is consistent with the observed clinical data [1]. In addition to combination therapy, the use of herbal supplements is continuously increasing. Recent studies disclosed that approximately 76% and 60% of adults in the USA and Europe, respectively, consume herbal supplements, and 25% of the herbal-drug consumers also take prescribed drugs [7,8], which increases the possibility of herb–drug interactions and may trigger the adverse effects of antihypertensive drugs. Adverse events of fimasartan were reported in 2.35% of patients. The most frequent events were dizziness (1.55%), headache (0.52%), abdominal pain, nausea, diarrhea, and coughing [9]. One case of angioedema was reported in 14,571 patients [9]. In addition, a case report described a 73-year-old patient with fimasartan-induced liver injury. Liver biopsy revealed hepatocellular necrosis and it was scored as a highly probable drug-induced liver injury [10,11].

Recently, an outcome study was conducted to evaluate the effects of early BP control and correction of metabolic abnormalities on future cardiovascular outcomes, relative to low-risk hypertension. Fimasartan reduced pulse pressure—a predictor of cardiovascular events—in the elderly (by −8.2 ± 0.3 mmHg) and nonelderly (by −7.0 ± 0.2 mmHg) (*p* < 0.0001), after adjusting for confounding factors, which indicates a higher efficacy in the elderly than that in the nonelderly [12,13]. Moreover, in a clinical study, “FANTASTIC”, to evaluate the rate of change in albuminuria in patients with diabetic chronic kidney disease, treatment with fimasartan for 6 months reduced albuminuria by more than 30%, which is expected to lower the risk of chronic kidney disease progression (compared with losartan). Based on these clinical outcomes, fimasartan can be recommended for patients with hypertension and chronic kidney disease [4,14].

**Figure 1:**
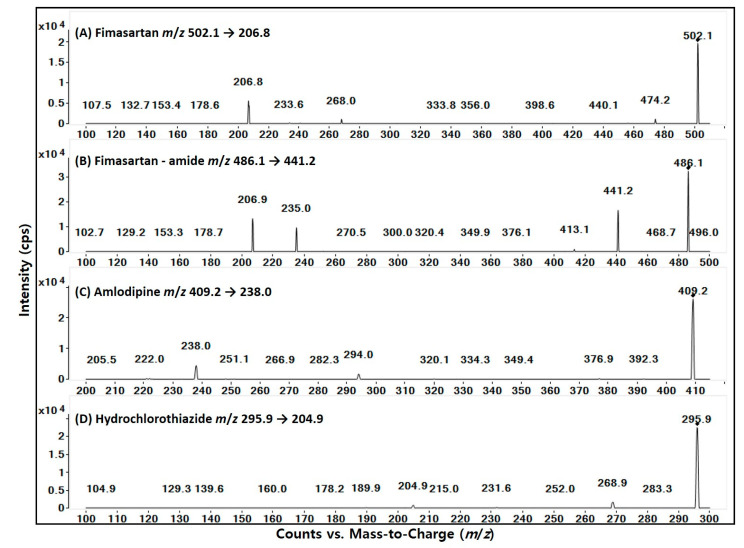
Structure and product ion mass spectra of (**A**) fimasartan, (**B**) fimasartan-amide, (**C**) amlodipine, and (**D**) hydrochlorothiazide.

The rapidly growing fimasartan market and combination trends may increase the risk of developing DDIs. Therefore, we aimed to develop sensitive and simple analytical methods to monitor the pharmacokinetic features of fimasartan, amlodipine, and hydrochlorothisazide, the most frequently prescribed combination. In addition, to understand the effect of fimasartan metabolism, we included an analytical method for the major and active metabolite of fimasartan, fimasartan-amide (oxidative desulfuration or BR-A-557, Figure 1) [15].

Red ginseng extract (RGE)—one of the most popular herbal medicines in many countries—has been reported for its anti-cancer, anti-diabetes, anti-inflammation, anti-oxidation, and liver protective effects. It also has adaptogenic effects in the modulation of immune function and cardiovascular functions [16,17,18]. Ginsenosides are responsible for the therapeutic efficacy of RGE [19,20,21] and, therefore, individual ginsenosides such as Rg3, compound K, protopanax diol, and protopanaxatriol and ginseng products have been under clinical evaluation or drug development process [7,16]. A meta-analysis [22,23,24] of the effect of RGE on the anti-hypertensive effect in five randomized clinical trials revealed significant acute effects of Korean red ginseng on systolic or diastolic blood pressure at 2–3 h, but failed to show long-term effects [25]. Rg3-enriched Korean ginseng products have shown remarkable anti-hypertensive effects in spontaneously hypertensive rats [26,27]. Co-administration of Rg3-enriched Korean ginseng and American ginseng was effective at reducing blood pressure, and favorable cardiometabolic outcomes were observed in 80 patients with type-2 diabetes (HbA1c: 6.5–8%) and hypertension (systolic BP: 140–160 mmHg) [28]. In addition to the beneficial effect of ginseng products with respect to the anti-hypertensive effect, the drug–drug interaction potential between ginseng and anti-hypertensive drugs needs to be evaluated as combination therapy with drugs having different modes of action is often prescribed, especially as therapeutic drugs are taken along with health supplements. Therefore, we aimed to investigate the pharmacokinetic interactions between RGE—an extract from dried root of ginseng of 6 years and more than 60% of dried ginseng content—and a triple anti-hypertensive drug combination (fimasartan, amlodipine, and hydrochlorothiazide) in rats.

## 2. Materials and Methods

### 2.1. Materials

RGE—an extract from dried root of ginseng of 6 years and more than 60% of dried ginseng content, 13.2 mg marker ginsenosides (GRb1 + GRg1 + GRg3), and 34.7 mg total ginsenosides per gram extract—was purchased from the Punggi Ginseng Cooperative Association (Youngjoo, Kyungpook, Korea) [29,30,31,32]. Amlodipine besylate, hydrochlorothiazide, and berberine hydrochloride (IS) were purchased from Sigma-Aldrich Chemical Co. (St. Louis, MO, USA). Fimasartan potassium trihydrate (fimasartan) and the oxidized desulfurized metabolite of fimasartan (fimasartan-amide) were obtained from Boryung Pharm. Co. Ltd. (Seoul, Korea). All other chemicals and solvents were of reagent or analytical grade.

### 2.2. LC-MS/MS Analysis and Validation of Fimasartan, Fimasartan-Amide, Amlodipine, and Hydrochlorothiazide

Fimasartan, fimasartan-amide, amlodipine, and hydrochlorothiazide levels in each sample were determined using an Agilent 6470 Triple Quadrupole LC-MS/MS system (Agilent, Wilmington, DE, USA). An isocratic mobile phase, which consisted of a mixture of water and acetonitrile (30:70, *v*/*v*) and contained 0.1% formic acid, was used at a flow rate of 0.20 mL/min to elute the analytes from the rat plasma matrix. Separation was performed using a Synergi Polar RP column (150 × 4.6 mm, 4 μm particle size; Pheonomenex, Torrance, CA, USA). The analytes were monitored using multiple reaction monitoring (MRM) mode, and the optimized mass conditions are listed in Table 1. Before starting sample analysis, the simultaneous analytical method was validated by evaluating the selectivity, linearity, accuracy, precision, recovery, and matrix effect. Three concentrations of quality control (QC) samples were prepared at low, medium, and high concentrations in the range of the standard calibration curves.

### 2.3. Analytical Validation

Calibration curves for a mixture of fimasartan, fimasartan-amide, amlodipine, and hydrochlorothiazide were prepared using the internal standard method. Briefly, aliquots (50 μL in acetonitrile) of seven different concentrations of the standard curve mixture that contained fimasartan (1, 2, 5, 20, 50, 200, and 500 ng/mL), fimasartan-amide (final concentrations of 0.3, 1, 2, 5, 20, 50, and 100 ng/mL), amlodipine (0.5, 2, 5, 20, 50, 100, and 200 ng/mL), and hydrochlorothiazide (5, 20, 50, 200, 500, 2000, and 5000 ng/mL) were dried under gentle nitrogen gas stream and reconstituted with 50 μL rat blank plasma. Then, 150 μL acetonitrile containing 1 ng/mL berberine (IS) was added to the reconstituted standard curves. After vortexing for 10 min and centrifuging for 10 min at 16,000× *g*, an aliquot (15 μL) of the supernatant was injected into the LC-MS/MS system. The linearity of the calibration standard was calculated from the peak height ratio of the analytes to the IS using the weight-adjusted method (1/x^2^). Three different concentrations of QC samples for fimasartan (3, 30, and 300 ng/mL), fimasartan-amide (0.5, 3, and 75 ng/mL), amlodipine (1.5, 15, and 150 ng/mL), and hydrochlorothiazide (15, 150, and 3000 ng/mL) were prepared according to the protocol described above.

The precision and accuracy of the inter- and intra-day assays were assessed by five or six measurements of three QC samples of fimasartan, fimasartan-amide, amlodipine, and hydrochlorothiazide. Precision was evaluated using the coefficient of variation (CV, %) of five or six QC sample measurements. Accuracy was calculated by dividing the measured QC concentration by the spiked QC concentration.

Extraction recovery was calculated by comparing the peak areas of the three QC samples in the pre-extraction samples with those of the post-extraction blank plasma spiked with the corresponding QC samples. Matrix effects were determined by comparing the peak area obtained from the post-extraction blank plasma spiked with three QC concentrations of the mixture of fimasartan, fimasartan-amide, amlodipine, and hydrochlorothiazide and the peak area from the corresponding concentration of the blank solution.

The stabilities of fimasartan, fimasartan-amide, amlodipine, and hydrochlorothiazide in rat plasma were tested under various conditions. Bench-top stability was calculated by comparing QC samples stored for 12 h at 25 °C with the untreated QC samples. For freeze–thaw stability, QC samples were analyzed after three freeze–thaw cycles. One cycle of the freeze–thaw process involved storing the QC samples at −80 °C for >12 h and thawing at 25 °C for 6 h. After three freeze–thaw cycles, the concentrations of the QC samples were determined using freshly prepared calibration standards. Post-preparative stability was evaluated by comparing the extracted QC samples maintained in the autosampler at 6 °C for 24 h (compared with the untreated QC samples).

### 2.4. Pharmacokinetic Study

Male Sprague-Dawley rats aged seven weeks (Samtako, Osan, Korea) were acclimatized for one week in an animal facility at Kyungpook National University. Food and water were provided ad libitum. All animal experiments were approved by the Institutional Animal Care and Use Committee of Kyungpook National University (approval no. KNU 2017-0044).

To investigate the DDIs among fimasartan, amlodipine, and hydrochlorothiazide, rats were randomly divided into a combination group (*n* = 6) and a single group (*n* = 6, for individual drug administration). The femoral arteries and veins of the rats were cannulated with PE50 polyethylene tubing (Jungdo, Seoul, Korea) under anesthesia with zoletil and lompun (50 and 5 mg/kg, respectively, intramuscular injection), and heparinized saline (10 U/mL) was used to prevent blood clotting. Pharmacokinetic studies were initiated after recovery from anesthesia. Each rat in the combination group received a mixture of fimasartan (3 mg/kg), amlodipine (5 mg/kg), and hydrochlorothiazide (5 mg/kg) dissolved in saline containing 10% DMSO. The rats in the single group orally received individual drug solutions at the same dose as present in the triple mixture solution. Blood samples were collected through the femoral artery at 0, 0.25, 0.5, 1, 2, 4, 8, and 24 h following the oral administration of fimasartan, amlodipine, and hydrochlorothiazide. After centrifugation of the blood samples at 8000× *g* for 1 min, 50 μL aliquots of the plasma samples were stored at −80 °C until analysis.

Rats were randomly divided into three groups, i.e., control (*n* = 6, vehicle treatment), single (*n* = 6, 1.5 g/kg), and multiple RGE treatments (*n* = 6, 1.5 g/kg for 7 days). Importantly, rats in the multiple RGE group received RGE suspension (1.5 g/kg/3 mL/day) for 7 days via oral gavage, while rats in the single RGE treatment group received water (3 mL/kg) for 6 days via oral gavage and RGE suspension (1.5 g/kg/3 mL) via oral gavage on the day 7. The control group received water (3 mL/kg) for seven days by oral gavage. One hour after the last RGE treatment, rats received a mixture of fimasartan, amlodipine, and hydrochlorothiazide at doses of 3, 5, and 5 mg/kg, respectively, by oral gavage. Subsequently, blood samples were collected via the femoral artery at 0, 0.25, 0.5, 1, 2, 4, 8, and 24 h. After centrifugation of the blood samples at 8000× *g* for 1 min, 50 μL aliquots of the plasma samples were stored at −80 °C until analysis.

Plasma samples (50 μL) were then mixed with 150 μL acetonitrile containing 1 ng/mL berberine (IS). After vortexing for 10 min and centrifuging for 10 min at 16,000× *g*, an aliquot (15 μL) of the supernatant was injected into the LC-MS/MS system.

### 2.5. Intestinal Permeability of Fimasartan, Amlodipine, and Hydrochlorothiazide

Randomly divided rats received vehicle (control group, *n* = 4), single (1.5 g/kg, *n* = 4), or multiple RGE treatments (1.5 g/kg for 7 days, *n* = 4). The procedure was the same as described previously. The jejunal segments were then isolated and rinsed using pre-warmed saline. Subsequently, they were mounted in the tissue holder of a Navicyte easy mount Ussing chamber (Warner Instruments, Holliston, MA, USA) and acclimated in Hank’s balanced salt solution (HBSS, pH 7.4) for 15 min with continuous oxygenation (95% O₂/5% CO₂). An intestinal permeability study was performed by adding 1 mL HBSS containing fimasartan, amlodipine, or hydrochlorothiazide (10 μM each) on the donor side and 1 mL fresh HBSS on the receiver side. Then, a 400 μL aliquot was withdrawn from the receiver side every 30 min for 2 h, and an equal volume of pre-warmed fresh HBSS was added to replenish the lost volume. For amlodipine analysis, 50 μL aliquots were mixed with 150 μL acetonitrile containing 1 ng/mL berberine (IS). After vortexing for 10 min and centrifuging for 10 min at 16,000× *g*, an aliquot (15 μL) of the supernatant was injected into the LC-MS/MS system.

### 2.6. Data Analysis and Statistics

Pharmacokinetic parameters were calculated using non-compartmental analyses and compared using the Kruskal–Wallis test. *p* < 0.05 was considered significant.

## 3. Results

### 3.1. Simultaneous Analysis of Fimasartan, Fimasartan-Amide, Amlodipine, and Hydrochlorothiazide

To optimize the electrospray ionization conditions for fimasartan, fimasartan-amide, amlodipine, and hydrochlorothiazide, each compound was directly injected into the mass spectrometer ionization source. The ionization mode and mass transition from Q1 to Q3 were selected based on the product ion scan results of authentic standards and previously published reports (Figure 1). Fimasartan, fimasartan-amide, and amlodipine had optimal ionization in the positive mode, whereas hydrochlorothiazide showed optimal ionization in the negative mode (Table 1). Optimized MRM transitions and MS/MS conditions for the analytes are listed in Table 1 and Figure 1, respectively. These conditions were consistent with those presented in previous reports [15,33,34,35,36].

**Figure 2:**
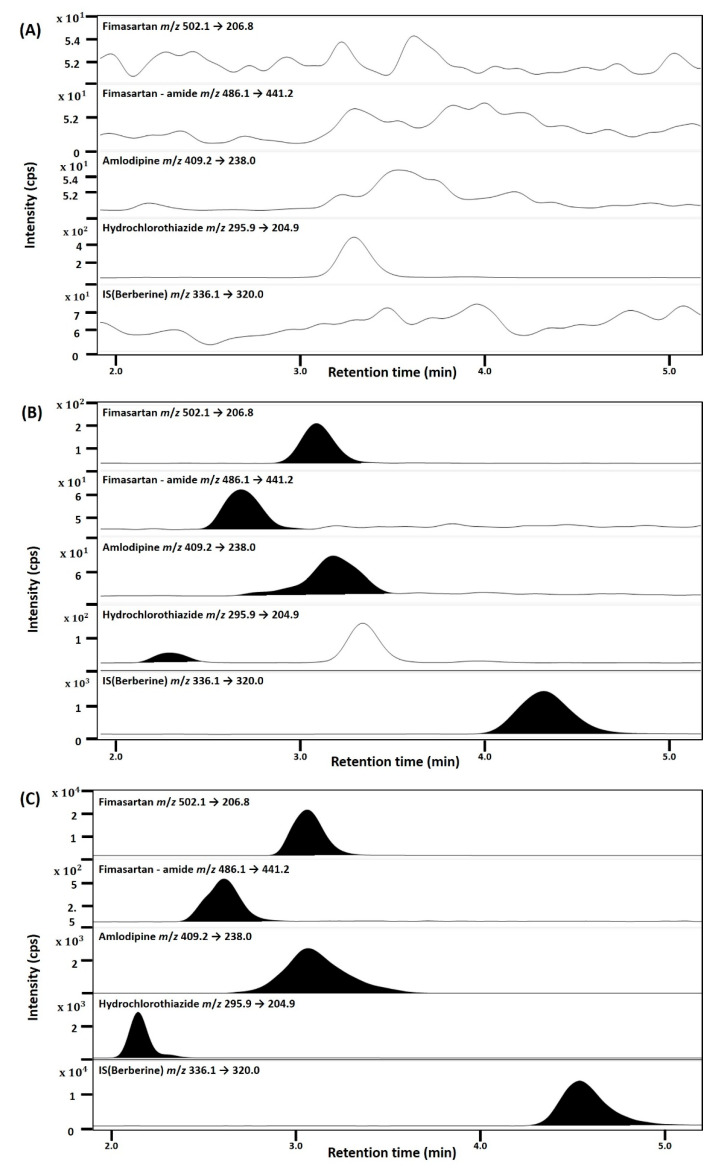
Representative multiple reaction monitoring (MRM) chromatogram of fimasartan, fimasartan-amide, amlodipine, hydrochlorothiazide, and IS (berberine) in (**A**) blank rat plasma; (**B**) rat blank plasma spiked with standard solution at a lower limit of quantification (LLOQ); and (**C**) rat plasma samples at 1 h following oral administration of fimasartan, amlodipine, and hydrochlorothiazide.

Selectivity was confirmed in male Sprague-Dawley rat plasma of eight different origins and was assessed by comparing the blank plasma peak response with that of plasma spiked with the lower limit of quantification (LLOQ) of fimasartan, fimasartan-amide, amlodipine, and hydrochlorothiazide (Table 1). The signal-to-noise ratios of each analyte of the LLOQ were all over 10. Figure 2 shows the chromatograms of the blank matrix and those of fimasartan, fimasartan-amide, amlodipine, and hydrochlorothiazide spiked at the LLOQ or rat plasma samples 1 h after oral administration of fimasartan, amlodipine, and hydrochlorothiazide. The peak retention times for fimasartan, fimasartan-amide, amlodipine, hydrochlorothiazide, and the IS were 3.1, 2.7, 3.2, 2.2, and 4.6 min, respectively. The results showed no disturbance peaks derived from rat blank plasma at the retention times of fimasartan, fimasartan-amide, amlodipine, hydrochlorothiazide, and IS under our MS/MS analysis conditions (Figure 2).

Linearity was evaluated using calibration curves of the mixture of fimasartan, fimasartan-amide, amlodipine, and hydrochlorothiazide (Table 1). The calibration curves showed good linearity in the ranges 1–500 ng/mL (for fimasartan), 0.3–100 ng/mL (for fimasartan-amide), 0.5–200 ng/mL (for amlodipine), and 5–5000 ng/mL (for hydrochlorothiazide), with a correlation coefficient (R^2^) of linear regression curves of >0.996 (Table 1).

Table 2 summarizes the intra- and inter-day precision and accuracy of fimasartan, fimasartan-amide, amlodipine, and hydrochlorothiazide. Precision was assessed as CV (%) for all analytes and the values were in the range of 2.38–6.77% in the intra-day batch and 1.96–7.75% in the inter-day batch. Accuracy is expressed as a percentage (%) of the concentrations determined from the calibration curve over the nominal concentrations. The accuracy of all analytes was in the range of 87.94–109.6% in the intra-day batch and 98.15–107.2% in the inter-day batch. The accuracy of the QC sample was obtained through back calculations from the equations of the standard curve. Owing to slight variations in the analytical process (e.g., changes in recovery during preparation of a biological sample and variability in the performance of the analytical instrument), there may be cases where the response of a QC sample is higher than that of a standard curve sample, but the inter- and intra-day accuracy in Table 2 were within the acceptance criteria for accuracy (i.e., 85~115%) according to the Guideline of Bioanalytical method validation from FDA, EMA, and ICH [37].

Table 3 summarizes the extraction recoveries and matrix effects. The extraction recoveries for fimasartan, fimasartan-amide, amlodipine, and hydrochlorothiazide were high and reproducible in the range of extraction recovery (75.92–96.73%) and CV (4.14–13.4%). Therefore, the protein precipitation method—employing three volumes of acetonitrile—utilized in this study could efficiently extract fimasartan, fimasartan-amide, amlodipine, and hydrochlorothiazide from rat plasma. The matrix effects ranged from 34.11% to 37.08% with a CV range of 9.44–13.6% for fimasartan, from 6.628% to 8.030% with a CV range of 8.90–10.9% for fimasartan-amide, from 39.87 to 49.35% with a CV range of 9.11–13.2% for amlodipine, and from 9.192% to 12.29% with a CV range of 6.73–11.1% for hydrochlorothiazide. The results suggested that the co-eluting substances showed substantial matrix effects on the ionization of the analytes. However, the matrix effects were stable for the three QC concentrations in the six samples of each plasma matrix. Therefore, we conclude that our sample preparation process can be used to analyze the concentrations of fimasartan, fimasartan-amide, amlodipine, and hydrochlorothiazide in rat plasma samples. The extraction recovery and matrix effects of the IS were also high and reproducible (Table 3).

The results of the stability experiments are presented in Table 4. The precision and accuracy for the bench-top stability, the post-preparative stability, and the three cycles of freeze–thaw stability were lower than 15%. These results provide evidence that fimasartan, fimasartan-amide, amlodipine, and hydrochlorothiazide in rat plasma samples were stable for up to 6 h at 25 °C (bench-top stability). Moreover, they were stable for 24 h in the autosampler tray after sample treatment and remained stable for three freeze–thaw cycles.

### 3.2. Comparative Pharmacokinetics of Fimasartan, Amlodipine, and Hydrochlorothiazide Following Oral Administration as Monotherapy or Combination Doses

**Figure 3:**
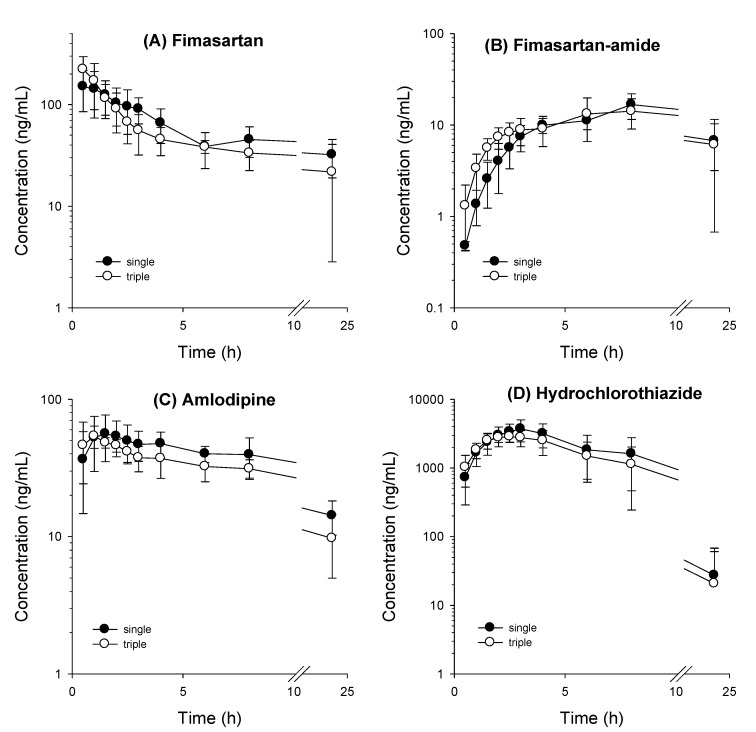
Plasma concentration–time profile of (**A**) fimasartan, (**B**) fimasartan-amide, (**C**) amlodipine, and (**D**) hydrochlorothiazide in rats that were orally administered a mixture of fimasartan (3 mg/kg), amlodipine (5 mg/kg), and hydrochlorothiazide (5 mg/kg) (triple, ●) or a single component with the same dose (single, ○). Data represent the mean ± standard deviation (*n* = 5).

First, we compared the pharmacokinetics of fimasartan, fimasartan-amide, amlodipine, and hydrochlorothiazide in rats, following their oral administration (as monotherapy or combination dose) (Figure 3). The plasma concentration profiles of fimasartan, fimasartan-amide, amlodipine, and hydrochlorothiazide were very similar, regardless of whether these drugs were orally administered individually or simultaneously as combination doses (3, 5, and 5 mg/kg for fimasartan, amlodipine, and hydrochlorothiazide, respectively). Pharmacokinetic parameters, such as C_max_, T_max_, AUC_last_, AUC_inf_, T_1/2_, and MRT values, of fimasartan, fimasartan-amide, amlodipine, and hydrochlorothiazide obtained following oral administration in the form of monotherapy were not significantly different from those obtained following simultaneous oral administration of a mixture of fimasartan, amlodipine, and hydrochlorothiazide (with corresponding doses) and were not significantly different among the three different RGE treatment groups (Table 5). The results suggest that concomitant use of a combination of fimasartan, amlodipine, and hydrochlorothiazide does not induce the development of pharmacokinetic DDI at oral doses of 3, 5, and 5 mg/kg, respectively, and we can further investigate the herb–drug interactions between RGE and triple combinations of the anti-hypertensive drugs at the current dose regimen.

### 3.3. Effect of RGE on the Pharmacokinetics of Fimasartan, Amlodipine, and Hydrochlorothiazide

**Figure 4:**
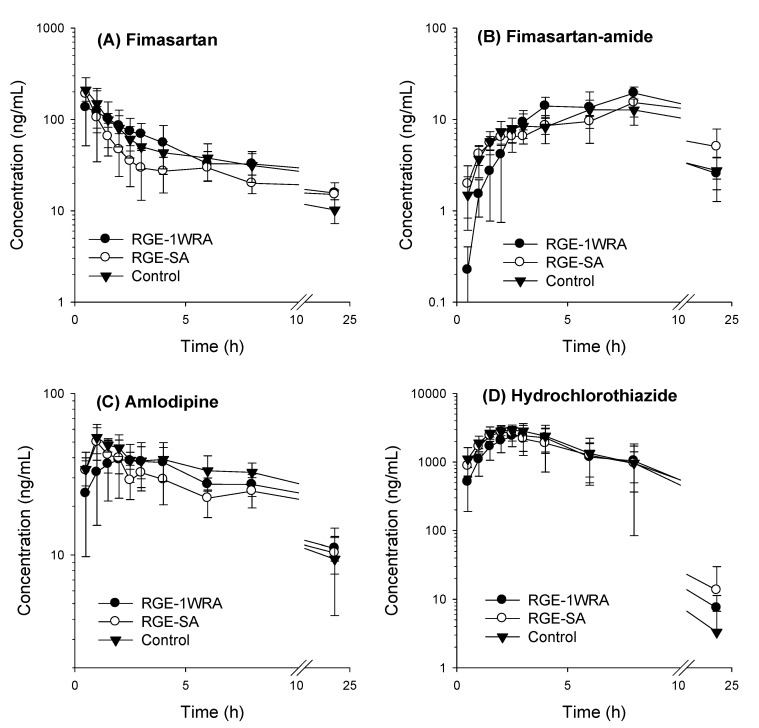
Plasma concentration–time profile of (**A**) fimasartan, (**B**) fimasartan-amide, (**C**) amlodipine, and (**D**) hydrochlorothiazide in rats orally administered a mixture of fimasartan (3 mg/kg), amlodipine (5 mg/kg), and hydrochlorothiazide (5 mg/kg) with vehicle (control group), single-dose RGE (1.5 g/kg; RGE-SA) and repeated-dose RGE (1.5 g/kg, once daily for one week; RGE-1WRA). Data represent the mean ± standard deviation (*n* = 5).

To investigate the herb–drug interactions between RGE and the antihypertensive drugs, the pharmacokinetic parameters of fimasartan, amlodipine, and hydrochlorothiazide in control rats were compared with those in rats that were orally administered a single dose of RGE and multiple doses of RGE for one week (Figure 4 and Table 6).

The plasma profile of fimasartan was similar, and pharmacokinetic parameters, such as C_max_, T_max_, AUC_last_, AUC_inf_, T_1/2_, and MRT values, of fimasartan were not significantly different among the three RGE treatment groups (Figure 4A and Table 6). Moreover, the plasma profile and pharmacokinetic parameters of fimasartan-amide, a major metabolite of fimasartan produced by CYP3A [34], were not significantly different among the three different RGE treatment groups (Figure 4B and Table 6). The results suggested that single or multiple administrations of RGE did not cause herb–drug interactions with fimasartan in terms of pharmacokinetics and metabolism. For amlodipine, C_max_, AUCl_ast_, AUC_inf_, T_1/2_, and MRT values were not significantly different between single and multiple administrations of RGE. However, the T_max_ of amlodipine was delayed by multiple administrations of RGE, but it was not affected by a single administration of RGE (*p* = 0.008 using the Kruskal–Wallis test, Figure 4C and Table 6). When comparing the plasma concentration profile of hydrochlorothiazide, no noticeable difference was detected between the different RGE treatment groups (Figure 4D). Pharmacokinetic parameters such as C_max_, T_max_, AUCl_ast_, AUC_inf_, T_1/2_, and MRT values of hydrochlorothiazide were not significantly different among the three RGE treatment groups (Table 6). Therefore, no pharmacokinetic herb–drug interaction was detected between RGE and hydrochlorothiazide. Taken together, all kinetic parameters except for the T_max_ of amlodipine were not significantly different among the different RGE treatment groups, suggesting a potential limited drug–drug interaction between RGE and the antihypertensive drug combination therapy.

**Figure 5:**
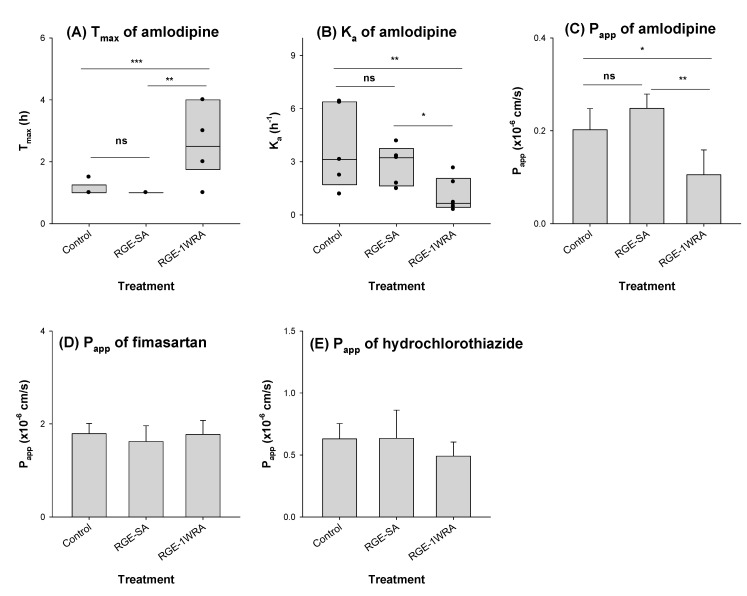
Comparison of (**A**) T_max_ and (**B**) K_a_ values of amlodipine in rats orally administered vehicle (control group), single-dose RGE (1.5 g/kg; RGE-SA), and repeated-dose RGE (1.5 g/kg, once daily for one week; RGE-1WRA). Permeability (P_app_) of (**C**) amlodipine, (**D**) fimasartan, and (**E**) hydrochlorothiazide in the jejunum of rats following the oral administration of vehicle (control group), single-dose RGE (1.5 g/kg; RGE-SA), and repeated-dose RGE (1.5 g/kg, once daily for one week; RGE-1WRA) was measured using the Ussing system. *p*-values were obtained from post-hoc analysis following the Kruskal–Wallis test. * *p* < 0.05, ** *p* < 0.01, and *** *p* < 0.001. Data represent the mean ± standard deviation (*n* = 5 for T_max_ and K_a_; *n* = 4 for permeability).

The T_max_ of amlodipine in the RGE multiple-treatment group was significantly higher than that in the other groups (i.e., control and RGE single-administration) (Figure 5A). As C_max_, AUC, T_1/2_, and MRT values were not altered by multiple RGE treatments, delayed T_max_ could reflect alterations in the absorption rate rather than the absorption extent. As expected, the absorption rate (K_a_) was significantly decreased in the RGE multiple-treatment group, but not significantly changed in the single-dose RGE group (Figure 5B). Amlodipine permeability was significantly decreased in the jejunal segments, which were isolated from rats with one-week-repeated administration of RGE groups (Figure 5C) and consistent with a decreased K_a_ value in repeated RGE administration.

To investigate whether the decreased permeability caused by repeated RGE treatment was specific for amlodipine, we also measured the intestinal permeability of fimasartan and hydrochlorothiazide. The P_app_ of fimasartan revealed moderate permeability and consistent with the faster T_max_. Moreover, the P_app_ of fimasartan was not affected by RGE treatment, either by single or multiple administrations (Figure 5D). For hydrochlorothiazide, the P_app_ was higher than that of amlodipine and was not affected by RGE treatment (Figure 5E). These results indicated that the decreased P_app_ by the repeated administration of RGE was specific to amlodipine among the combination drugs and that it may decrease the absorption rate of this drug.

## 4. Discussion

Fimasartan is effective at reducing blood pressure. In large clinical studies, fimasartan showed an excellent safety profile and, when combined with hydrochlorothiazide or amlodipine, it showed a better effect with respect to controlling blood pressure than that of monotherapy. It has beneficial effects with respect to protecting against major adverse cardiovascular events and its renoprotective effects in hypertensive diabetic chronic kidney disease are under evaluation [4,12,14,38]. Preclinical studies have demonstrated organ-protective effects of fimasartan [4]. These results suggest that fimasartan is an attractive candidate for the treatment of hypertension and suggest the increasing use of fimasartan monotherapy and combination therapy. Fimasartan and its combination drugs have been introduced into the market in form of the following formulations: Kanarb^®^ (fimasartan), Kanarb plus^®^ (fimasartan and hydrochlorothiazide), Tovero^®^ (fimasartan and rosuvastatin), Dukaro^®^ (fimasartan, amlodipine, and rosuvastatin), Akarb^®^ (fimasartan and atorvastatin), and Dukarb plus^®^ (fimasartan, amlodipine, and hydrochlorothiazide). With the increasing use of fimasartan and its combination formulation, the development of analytical methods for frequently used combinations of antihypertensive drugs is crucial to investigate the pharmacokinetic studies of these drugs.

Jung et al. [6] investigated the pharmacokinetic interactions of triple combinations of fimasartan, amlodipine, and hydrochlorothiazide. For this, they applied two different sample preparation methods including protein precipitation for fimasartan/amlodipine and liquid–liquid extraction for hydrochlorothiazide. In this study, we developed simple and simultaneous analytical method for fimasartan, fimasartan-amide (a major metabolite of fimasartan), amlodipine, and hydrochlorothiazide in rat plasma using the protein precipitation method; therefore, it has advantages of a simple sample pretreatment procedures and a shortened run time. In addition, we included the analysis of a major active metabolite of fimasartan, fimasartan-amide, which is expected to be easily applied to efficacy and pharmacokinetic-pharmacodynamic studies of antihypertensive combination drugs as well. Finally, to the best of our knowledge, this is the first validated report of an LC-MS/MS method for the simple and simultaneous determination of fimasartan, fimasartan-amide, amlodipine, and hydrochlorothiazide from rat plasma.

A number of analytical methods for monitoring individual fimasartan, amlodipine, or hydrochlorothiazide in plasma samples using tandem mass did not use the quantitative and qualitative MRM trace. We also used the same MRM condition as previously published reports [39,40] and the product ion mass spectra from fimasartan, amlodipine, and hydrochlorothiazide showed different mass fragmentation patterns (Figure 1). In addition, in a selectivity evaluation, we confirmed that there were no interfering peaks in the individual substance compared with all four substances spiked in blank rat plasma. However, the use of two MRM traces would have increased the quality and usability of the analytical method, as it provides more certainty in identifying the analytes among the existing exogenous and endogenous structural analogues [41]. Therefore, it is necessary to apply and validate two MRM trace methods for fimasartan and fimasartan-amide, amlodipine, and hydrochlorothiazide in our future analysis.

We then applied our analytical method to evaluate the pharmacokinetic interactions among the triple combination of these drugs and the effect of RGE treatment on the same. Pharmacokinetic interactions among the triple combination drugs were evaluated by comparing oral monotherapy of fimasartan, amlodipine, and hydrochlorothiazide, as well as the oral administration of the triple mixture with the corresponding dose. The results indicated no interaction in the pharmacokinetic parameters of the orally administered triple combination compared with those of the individual components (Figure 3 and Table 5). When compared with the previous pharmacokinetic results of antihypertensive drugs, pharmacokinetic parameters such as C_max_, T_max_, AUC, and T_1/2_ of fimasartan in this study were similar to those in rats with the same dose regimen (PO, 3 mg/kg) [33]. In the case of amlodipine, we could not find the pharmacokinetic profile of amlodipine in rats with the same dose regimen. Considering that amlodipine has a linear pharmacokinetic profile, with a positive correlation between oral dosage and C_max_ and AUC [42], dose-normalized AUC values of amlodipine were in the range of 181–349 ng∙h/mL/(mg/kg oral dose) [35,42], which was similar to our case (159–203 ng∙h/mL/(mg/kg oral dose). T_1/2_ of amlodipine (10.22–13.46 h in this study) was also similar to the previous reports (6.3–13.03 h) [35,42]. Pharmacokinetic parameters such as T_1/2_ and the volume of the distribution of hydrochlorothiazide showed dose linearity in the oral dose range of 7.5–30 mg/kg [43]. In this regard, Asdaq et al. reported dose-normalized AUC values of 4.69 μg∙h/mL/(mg/kg oral dose) following its oral administration (10 mg/kg) [44]. Consistently, our results yielded dose-normalized AUC values of 4.58 μg∙h/mL/(mg/kg oral dose) after an oral dose of 5 mg/kg (Table 4). These results suggested that our pharmacokinetic parameters for fimasartan, amlodipine, and hydrochlorothiazide were comparable to the previously reported values and triple combination of antihypertensive drugs. Therefore, fimasartan, amlodipine, and hydrochlorothiazide could be concomitantly administered at doses of 3 mg/kg, 5 mg/kg, and 5 mg/kg, respectively, for the future investigation of pharmacokinetic interaction, efficacy, and toxicokinetic studies.

Next, we evaluated the interactions between RGE and the triple combination. No significant difference was observed in the pharmacokinetic parameters of fimasartan, fimasartan-amide, amlodipine, and hydrochlorothiazide (except for the T_max_ of amlodipine) (Figure 4 and Table 6). The delayed T_max_ of amlodipine is consistent with the previous results of Ryu et al. [35]. As C_max_, AUC, T_1/2_, and MRT values of amlodipine were not altered by multiple RGE treatments, delayed T_max_ could reflect alterations in the absorption rate rather than the absorption extent and the excretion rate. Consistently, the absorption rate (K_a_) and the absorption permeability (P_app_) in the rat intestine following repeated RGE treatment were significantly decreased compared with control group as well as single RGE treatment (Figure 5). Taken together, the delayed T_max_ could be attributed to the decreased intestinal permeability and decreased absorption rate of amlodipine by repeated RGE administration at a dose of 1.5 g/kg/day. In addition, the decreased intestinal permeability by the repeated administration of RGE was specific to amlodipine among three combination drugs. The mechanism for the RGE treatment-dependent change in the absorption rate of amlodipine, but not fimasartan and hydrochlorothiazide, is unclear in the present study. However, as P_app_ of amlodipine was nine- and threefold lower than that of fimasartan and hydrochlorothiazide, respectively (Figure 5), the limited and decreased intestinal permeability of amlodipine as well as physiological changes in the intestine by the repeated administration of RGE may alter the absorption rate of amlodipine [45].

## 5. Conclusions

Our validated analytical method for fimasartan, its active metabolite fimasartan-amide, amlodipine, and hydrochlorothiazide can be easily extrapolated to pharmacokinetic, efficacy, and toxicokinetic studies. In an application study, we successfully assessed the pharmacokinetic interactions between these triple combination drugs and co-administered drugs and herbal supplements. In addition, repeated administration of RGE (1.5 g/kg/day) for one week delayed the absorption of amlodipine compared with single RGE administration (or the control group), but no interaction was detected between hydrochlorothiazide and fimasartan. In conclusion, using a triple combination of anti-hypertensive drugs and simultaneous analytical methods, we conducted efficient drug interaction and pharmacokinetic studies using fewer animals.

## Figures and Tables

**Table 1:** MS/MS parameters for the detection and standard calibration curves for fimasartan, fimasartan-amide, amlodipine, and hydrochlorothiazide.

Compounds	Fimasartan	Fimasartan-Amide	Amlodipine	Hydrochlorothiazide
MRM Transitions (*m*/*z*)	502.1 → 206.8	486.1 → 441.2	409.2 → 238.0	295.9 → 204.9
Ionization Mode	positive	positive	positive	negative
Fragmentwr (V)	125	135	105	125
Collision Energy (eV)	25	10	8	20
Linear Range (ng/mL)	1–500	0.3–100	0.5–200	5–5000
LLOQ (ng/mL)	1	0.3	0.5	5
Representative Equation	Y = 0.03874x + 0.002912	Y = 0.00978 + 0.000351	Y = 0.01158x + 0.002238	Y = 0.00083x + 0.000086
R^2^	0.9998	0.9992	0.9964	0.9975

**Table 2:** Intra- and inter-day precision and accuracy of fimasartan, fimasartan-amide, amlodipine, and hydrochlorothiazide.

Analytes	Nominal Concentration (ng/mL)	Intra-Day (*n* = 5)			Inter-Day (*n* = 6)		
		Measured Concentration (ng/mL)	Precision (CV, %)	Accuracy (%)	Measured Concentration (ng/mL)	Precision (CV, %)	Accuracy (%)
Fimasartan	3	3.265	2.38	108.8	3.207	3.10	106.9
	30	32.44	4.56	108.1	30.50	5.47	101.7
	300	316.1	3.81	105.4	318.8	5.38	106.3
Fimasartan-amide	0.5	0.528	6.77	105.6	0.528	5.28	105.5
	3	3.263	2.73	108.8	3.172	3.79	105.7
	75	70.54	4.24	94.06	74.04	3.43	98.72
Amlodipine	1.5	1.644	4.17	109.6	1.573	7.75	104.9
	15	15.99	5.43	106.6	15.36	2.46	102.4
	150	159.3	3.23	106.2	149.6	1.96	99.73
Hydrochlorothiazide	15	13.99	5.06	93.24	15.14	4.09	100.9
	150	131.9	3.00	87.94	147.2	5.58	98.15
	3000	2937	4.51	97.91	3215	4.68	107.2

Data represented as mean and CV from five or six independent experiments.

**Table 3:** Extraction recoveries and matric effects for the determination of fimasartan, fimasartan-amide, amlodipine, and hydrochlorothiazide.

Analyte	Concentration (ng/mL)	Extraction Recovery (%)	CV (%)	Matrix Effects (%)	CV (%)
Fimasartan	3	90.30	10.6	35.06	13.6
	30	75.92	8.59	37.08	13.3
	300	77.62	13.4	34.11	9.44
Fimasartan-amide	0.5	91.91	4.98	6.628	9.53
	3	89.43	4.14	6.839	8.90
	75	96.73	6.53	8.030	10.9
Amlodipine	1.5	85.55	6.77	49.35	9.30
	15	80.00	4.83	47.18	9.11
	150	89.02	8.82	39.87	13.2
Hydrochlorothiazide	15	93.44	5.85	12.29	6.73
	150	93.88	9.25	9.192	11.1
	3000	95.19	11.7	11.17	6.64
IS	1	97.25	7.21	89.26	6.84

Data represented as mean and CV from six independent experiments.

**Table 4:** Stability of fimasartan, fimasartan-amide, amlodipine, and hydrochlorothiazide.

Storage Conditions	Analytes	Concentration (ng/mL)		Precision (CV%)	Accuracy (%)
		Spiked	Measured		
Bench-top stability (6 h at 25 °C)	Fimasartan	3	2.86	1.09	95.42
		300	298.4	4.12	99.48
	Fimasartan-amide	0.5	0.55	3.63	110.6
		75	72.24	4.87	96.32
	Amlodipine	1.5	1.47	2.67	98.31
		150	157.6	3.49	105.1
	Hydrochlorothiazide	15	13.40	4.53	89.34
		3000	2864	1.16	95.47
Post-preparative stability (24 h at 6 °C)	Fimasartan	3	3.21	4.96	106.9
		300	293.1	6.11	97.70
	Fimasartan-amide	0.5	0.56	6.28	111.2
		75	71.14	3.74	94.86
	Amlodipine	1.5	1.41	7.27	94.04
		150	150.3	6.65	100.2
	Hydrochlorothiazide	15	14.36	8.42	95.73
		3000	2973	9.40	99.11
Three freeze–thaw cycle stability	Fimasartan	3	3.22	2.81	107.4
		300	322.6	4.17	107.5
	Fimasartan-amide	0.5	0.56	0.35	111.0
		75	78.53	5.84	104.7
	Amlodipine	1.5	1.53	1.89	101.7
		150	166.8	2.43	111.2
	Hydrochlorothiazide	15	13.21	3.60	88.08
		3000	3118	6.74	103.9

Data represented as mean and CV from three independent experiments.

**Table 5:** Pharmacokinetic parameters of fimasartan, fimasartan-amide, amlodipine, and hydrochlorothiazide in rats that were orally administered monotherapy or a combination of fimasartan (3 mg/kg), amlodipine (5 mg/kg), and hydrochlorothiazide (5 mg/kg).

Drugs	PK Parameters	Single	Triple	*p*-Value
Fimasartan	C_max_ (ng/mL)	170.1 ± 51.7	222.3 ± 73.9	0.240
	T_max_ (h)	1.7 ± 1.4	0.5 ± 0.0	0.065
	AUC_last_ (ng∙h/mL)	1218 ± 253	994.8 ± 367	0.589
	AUC_inf_ (ng∙h/mL)	2219 ± 591	1344 ± 868	0.126
	T_1/2_ (h)	22.1 ± 12.3	23.0 ± 25.2	0.631
	MRT (h)	8.9 ± 1.5	7.5 ± 1.9	0.093
Fimasartan-amide	C_max_ (ng/mL)	16.9 ± 5.1	17.0 ± 4.8	0.699
	T_max_ (h)	7.7 ± 0.8	10.0 ± 4.8	0.923
	AUC_last_ (ng∙h/mL)	254.3 ± 68.6	236.1 ± 82.3	0.522
	AUC_inf_ (ng∙h/mL)	NC	NC	
	T_1/2_ (h)	NC	NC	
	MRT (h)	10.6 ± 1.5	10.4 ± 1.5	0.394
	MR	0.2	0.2	
Amlodipine	C_max_ (ng/mL)	64.9 ± 15.4	61.6 ± 11.5	0.310
	T_max_ (h)	1.4 ± 0.9	1.1 ± 0.7	0.394
	AUC_last_ (ng∙h/mL)	699.7 ± 249	625.2 ± 103	0.485
	AUC_inf_ (ng∙h/mL)	1018 ± 269	798.8 ± 197	0.065
	T_1/2_ (h)	11.3 ± 1.9	10.7 ± 4.8	0.818
	MRT (h)	7.8 ± 1.8	7.9 ± 0.9	0.589
Hydrochlorothiazide	C_max_ (ng/mL)	3785 ± 1263	3289 ± 391	0.423
	T_max_ (h)	3.3 ± 0.6	2.8 ± 1.0	0.406
	AUC_last_ (μg∙h/mL)	31.5 ± 15.8	24.7 ± 11.1	0.423
	AUC_inf_ (μg∙h/mL)	31.6 ± 15.9	24.8 ± 11.1	0.423
	T_1/2_ (h)	2.7 ± 0.7	2.6 ± 1.2	0.262
	MRT (h)	5.5 ± 0.7	5.1 ± 1.0	0.522

Data are represented as mean ± standard deviation from six rats; NC: not calculated; C_max_: maximum plasma concentration; AUC_last or_ AUC_inf_: area under the plasma concentration–time curve from zero to last time or infinity; T_max_, time to reach C_max_; T_1/2_, elimination half-life; MRT, mean residence time. The *p*-value was calculated using the non-parametric Mann–Whitney U test.

**Table 6:** Pharmacokinetic parameters of fimasartan, fimasartan-amide, amlodipine, and hydrochlorothiazide in rats orally administered a mixture of fimasartan (3 mg/kg), amlodipine (5 mg/kg), and hydrochlorothiazide (5 mg/kg) with vehicle (control group), single-dose RGE (1.5 g/kg; RGE-SA), and repeated-dose RGE (1.5 g/kg, once daily for one week; RGE-1WRA).

Drugs	PK Parameters	RGE Treatment (1.5 g/kg)			*p*-Value
		Control	RGE-SA	RGE-1WRA	
Fimasartan	C_max_ (ng/mL)	210.1 ± 756	190.1 ± 32.4	147.2 ± 72.3	0.181
	T_max_ (h)	0.50 ± 0.00	0.50 ± 0.00	1.25 ± 1.41	0.169
	AUC_last_ (ng∙h/mL)	840.9 ± 258.9	643.3 ± 122.0	903.5 ± 299.9	0.075
	AUC_inf_ (ng∙h/mL)	999.9 ± 268.9	1245.3 ± 334.5	1284.6 ± 314.4	0.336
	T_1/2_ (h)	10.68 ± 3.33	21.63 ± 4.77	22.06 ± 16.7	0.104
	MRT (h)	7.47 ± 0.60	8.05 ± 0.42	7.93 ± 0.58	0.105
Fimasartan-amide	C_max_ (ng/mL)	15.80 ± 4.82	15.32 ± 4.71	19.93 ± 2.81	0.181
	T_max_ (h)	7.20 ± 1.10	8.00 ± 0.00	7.33 ± 1.63	0.343
	AUC_last_ (ng∙h/mL)	193.3 ± 52.7	227.2 ± 50.9	257.8 ± 30.2	0.135
	AUC_inf_ (ng∙h/mL)	235.6 ± 93.9	NC	NC	NC
	T_1/2_ (h)	6.18 ± 1.32	NC	NC	NC
	MRT (h)	8.69 ± 0.71	9.98 ± 1.60	8.49 ± 0.82	0.294
	MR	0.24 ± 0.10	0.37 ± 0.12	0.31 ± 0.11	0.120
Amlodipine	C_max_ (ng/mL)	54.38 ± 8.96	49.97 ± 11.4	48.50 ± 10.5	0.560
	T_max_ (h)	1.10 ± 0.22	1.00 ± 0.00	2.67 ± 1.21	0.008
	AUC_last_ (ng∙h/mL)	630.5 ± 108.2	515.4 ± 95.8	557.3 ± 74.5	0.279
	AUC_inf_ (ng∙h/mL)	795.6 ± 218.4	788.5 ± 247.2	774.8 ± 82.1	0.940
	T_1/2_ (h)	10.22 ± 5.09	12.44 ± 1.96	13.46 ± 2.91	0.138
	MRT (h)	7.90 ± 0.93	8.62 ± 0.88	8.75 ± 0.94	0.319
Hydrochlorothiazide	C_max_ (ng/mL)	3309.8 ± 433.4	3071.6 ± 583.1	2668.9 ± 861.2	0.426
	T_max_ (h)	2.60 ± 0.96	2.30 ± 0.97	3.42 ± 0.66	0.101
	AUC_last_ (μg∙h/mL)	22.90 ± 11.4	20.90 ± 7.96	20.91 ± 9.92	0.940
	AUC_inf_ (μg∙h/mL)	23.05 ± 11.4	21.01 ± 8.06	21.01 ± 10.0	0.916
	T_1/2_ (h)	2.65 ± 1.29	2.77 ± 0.64	2.71 ± 0.65	0.560
	MRT (h)	4.89 ± 1.03	5.29 ± 0.60	5.47 ± 0.66	0.426

Data are represented as mean ± standard deviation from five rats; NC: not calculated; C_max_: maximum plasma concentration; AUC_last_ or AUC_inf_: area under the plasma concentration–time curve from zero to last time or infinity; T_max_, time to reach C_max_; T_1/2_, elimination half-life; MRT, mean residence time. The *p*-value was calculated using the non-parametric Kruskal–Wallis test.

## Data Availability

Not applicable.

## References

[1] Rhee S.J., Lee H.A., Lee S., Kim E., Jeon I., Song I.S., Yu K.S. (2018). Physiologically based pharmacokinetic modeling of fimasartan, amlodipine, and hydrochlorothiazide for the investigation of drug–drug interaction potentials. Pharm. Res..

[2] Rhee M.Y., Baek S.H., Kim W., Park C.G., Park S.W., Oh B.H., Kim S.H., Kim J.J., Shin J.H., Yoo B.S. (2015). Efficacy of fimasartan/hydrochlorothiazide combination in hypertensive patients inadequately controlled by fimasartan monotherapy. Drug Des. Devel. Ther..

[3] Yi S., Kim T.E., Yoon S.H., Cho J.Y., Shin S.G., Jang I.J., Yu K.S. (2011). Pharmacokinetic interaction of fimasartan, a new angiotensin II receptor antagonist, with amlodipine in healthy volunteers. J. Cardiovasc. Pharmacol..

[4] Lee H.Y., Oh B.H. (2016). Fimasartan: A new angiotensin receptor blocker. Drugs.

[5] Jeon H., Lim K.S., Shin K.H., Kim J., Yoon S.H., Cho J.Y., Shin S.G., Jang I.J., Yu K.S. (2012). Assessment of the drug-drug interactions between fimasartan and hydrochlorothiazide in healthy volunteers. J. Cardiovasc. Pharmacol..

[6] Jung J., Lee S., Oh J., Lee S., Jang I.-J., Lee D., Yu K.S. (2021). Pharmacokinetic comparison between a fixed-dose combination of fimasartan/amlodipine/hydrochlorothiazide 60/10/25 mg and a corresponding loose combination of fimasartan/amlodipine 60/25 mg and hydrochlorothiazide 25 mg in healthy subjects. Transl. Clin. Pharmacol..

[7] Choi M.K., Song I.S. (2019). Interactions of ginseng with therapeutic drugs. Arch. Pharm. Res..

[8] Jeurissen S.M.F., Buurma-Rethans E.J.M., Beukers M.H., Jansen-van der Vliet M., van Rossum C.T.M., Sprong R.C. (2018). Consumption of plant food supplements in the Netherlands. Food Funct..

[9] Park J.B., Sung K.-C., Kang S.-M., Cho E.J. (2013). Safety and efficacy of fimasartan in patients with arterial hypertension (Safe-KanArb Study). Am. J. Cardiovas. Drugs.

[10] Park D.H., Yun G.Y., Eun H.S., Joo J.S., Kim J.S., Kang S.H., Moon H.S., Lee E.S., Lee B.S., Kim K.H. (2017). Fimasartan-induced liver injury in a patient with no adverse reactions on other types of angiotensin II receptor blockers: A case report. Medicine.

[11] Real M., Barnhill M.S., Higley C., Rosenberg J., Lewis J.H. (2019). Drug-induced liver injury: Highlights of the recent literature. Drug Saf..

[12] Cho E.J., Sung K.C., Kang S.M., Shin M.S., Joo S.J., Park J.B. (2019). Fimasartan reduces clinic and home pulse pressure in elderly hypertensive patients: A K-MetS study. PLoS ONE.

[13] Kim C., Kim M.Y., Kang D.R., Kim J.Y., Park J.B. (2014). The efficacy of fimasartan for cardiovascular events and metabolic syndrome (K-MetS Study): Rationale, design and participant characteristics. Pulse.

[14] Kim J.Y., Son J.W., Park S., Yoo T.H., Kim Y.J., Ryu D.R., Chin H.J. (2017). FimAsartaN proTeinuriA SusTaIned reduCtion in comparison with losartan in diabetic chronic kidney disease (FANTASTIC): Study protocol for randomized controlled trial. Trials..

[15] Shin B.S., Kim T.H., Paik S.H., Chi Y.H., Lee J.H., Tan H.K., Choi Y., Kim M., Yoo S.D. (2011). Simultaneous determination of fimasartan, a novel antihypertensive agent, and its active metabolite in rat plasma by liquid chromatography-tandem mass spectrometry. Biomed. Chromatogr..

[16] Jeon J.H., Lee J., Park J.H., Lee C.H., Choi M.K., Song I.S. (2021). Effect of lactic acid bacteria on the pharmacokinetics and metabolism of ginsenosides in mice. Pharmaceutics.

[17] Irfan M., Kwak Y.S., Han C.K., Hyun S.H., Rhee M.H. (2020). Adaptogenic effects of Panax ginseng on modulation of cardiovascular functions. J. Ginseng Res..

[18] Ratan Z.A., Youn S.H., Kwak Y.S., Han C.K., Haidere M.F., Kim J.K., Min H., Jung Y.J., Hosseinzadeh H., Hyun S.H. (2021). Adaptogenic effects of Panax ginseng on modulation of immune functions. J. Ginseng Res..

[19] Leung K.W., Wong A.S. (2010). Pharmacology of ginsenosides: A literature review. Chin. Med..

[20] Kim J.H., Yi Y.S., Kim M.Y., Cho J.Y. (2017). Role of ginsenosides, the main active components of Panax ginseng, in inflammatory responses and diseases. J. Ginseng Res..

[21] Gui Q.F., Xu Z.R., Xu K.Y., Yang Y.M. (2016). The efficacy of ginseng-related therapies in type 2 diabetes mellitus: An updated systematic review and meta-analysis. Medicine.

[22] Lee H.W., Lim H.J., Jun J.H., Choi J., Lee M.S. (2017). Ginseng for Treating Hypertension: A systematic review and meta-analysis of double blind, randomized, placebo-controlled trials. Curr. Vasc. Pharmacol..

[23] Park S.H., Chung S., Chung M.-Y., Choi H.-K., Hwang J.-T., Park J.H. (2022). Effects of panax ginseng on hyperglycemia, hypertension, and hyperlipidemia: A systematic review and meta-analysis. J. Ginseng Res..

[24] Hur M.-H., Lee M.-S., Yang H.-J., Kim C., Bae I.-L., Ernst E. (2010). Ginseng for reducing the blood pressure in patients with hypertension: A systematic review and meta-analysis. J. Ginseng Res..

[25] Stavro P.M., Woo M., Heim T.F., Leiter L.A., Vuksan V. (2005). North American ginseng exerts a neutral effect on blood pressure in individuals with hypertension. Hypertension.

[26] Lee K.H., Bae I.Y., Park S.I., Park J.-D., Lee H.G. (2016). Antihypertensive effect of Korean Red Ginseng by enrichment of ginsenoside Rg3 and arginine–fructose. J. Ginseng Res..

[27] Nagar H., Kang S.K., Choi S.W., Song H.-J., Choi S.-J., Piao S., Kim S., Lee I., Kim C.-S. (2020). Antihypertensive effects of Rg3-enriched Korean vitamin ginseng in spontaneously hypertensive rats. Nat. Prod. Commun..

[28] Jovanovski E., Smircic-Duvnjak L., Komishon A., Au-Yeung F.R., Sievenpiper J.L., Zurbau A., Jenkins A.L., Sung M.-K., Josse R., Li D. (2021). Effect of coadministration of enriched Korean Red Ginseng (Panax ginseng) and American ginseng (Panax quinquefolius L) on cardiometabolic outcomes in type-2 diabetes: A randomized controlled trial. J. Ginseng Res..

[29] Jeon J.H., Lee S., Lee W., Jin S., Kwon M., Shin C.H., Choi M.K., Song I.S. (2020). Herb-drug interaction of red ginseng extract and ginsenoside Rc with valsartan in rats. Molecules.

[30] Jin S., Lee S., Jeon J.H., Kim H., Choi M.K., Song I.S. (2019). Enhanced intestinal permeability and plasma concentration of metformin in rats by the repeated administration of red ginseng extract. Pharmaceutics.

[31] Lee S., Kwon M., Choi M.K., Song I.S. (2018). Effects of red ginseng extract on the pharmacokinetics and elimination of methotrexate via Mrp2 regulation. Molecules.

[32] Nam S.J., Han Y.J., Lee W., Kang B., Choi M.K., Han Y.H., Song I.S. (2018). Effect of red ginseng extract on the pharmacokinetics and efficacy of metformin in streptozotocin-induced diabetic rats. Pharmaceutics.

[33] Kim T.H., Shin S., Bashir M., Chi Y.H., Paik S.H., Lee J.H., Choi H.J., Choi J.H., Yoo S.D., Bulitta J.B. (2014). Pharmacokinetics and metabolite profiling of fimasartan, a novel antihypertensive agent, in rats. Xenobiotica.

[34] Choi Y.J., Lee J.Y., Ryu C.S., Chi Y.H., Paik S.H., Kim S.K. (2018). Role of cytochrome P450 enzymes in fimasartan metabolism in vitro. Food Chem. Toxicol..

[35] Ryu S.H., Kim J.W., Kim Y.S., Lee S.-H., Cho Y.-B., Lee H.K., Kim Y.G., Jeong W.-S., Kim K.-B. (2014). Negligible pharmacokinetic interaction of red ginseng and antihypertensive agent amlodipine in Sprague-Dawley rats. J. Toxicol. Environ. Health Part A.

[36] Shah J.V., Parekh J.M., Shah P.A., Shah P.V., Sanyal M., Shrivastav P.S. (2017). Application of an LC–MS/MS method for the analysis of amlodipine, valsartan and hydrochlorothiazide in polypill for a bioequivalence study. J. Pharm. Anal..

[37] Kaza M., Karaźniewicz-Łada M., Kosicka K., Siemiątkowska A., Rudzki P.J. (2019). Bioanalytical method validation: New FDA guidance vs. EMA guideline. Better or worse?. J. Pharm. Biomed. Anal..

[38] Pradhan A., Gupta V., Sethi R. (2019). Fimasartan: A new armament to fight hypertension. J. Family Med. Prim. Care.

[39] Yang E., Lee S., Lee H., Hwang I., Jang I.J., Yu K.S., Lee S. (2019). Pharmacokinetic comparison between fixed-dose combination of fimasartan/amlodipine 60/10 mg and the corresponding loose combination through partial replicated crossover study in healthy subjects. Transl. Clin. Pharmacol..

[40] Kumar A., Dwivedi S.P., Prasad T. (2019). Method validation for simultaneous quantification of olmesartan and hydrochlorothiazide in human plasma using LC-MS/MS and its application through bioequivalence study in healthy volunteers. Front. Pharmacol..

[41] Madureira T.V., Barreiro J.C., Rocha M.J., Cass Q.B., Tiritan M.E. (2009). Pharmaceutical trace analysis in aqueous environmental matrices by liquid chromatography–ion trap tandem mass spectrometry. J. Chromatogr. A.

[42] Gadepalli S.G., Deme P., Kuncha M., Sistla R. (2014). Simultaneous determination of amlodipine, valsartan and hydrochlorothiazide by LC-ESI-MS/MS and its application to pharmacokinetics in rats. J. Pharm. Anal..

[43] Hao K., Chen Y., Zhao X., Liu X. (2014). Pharmacokinetic-pharmacodynamic model of the antihypertensive interaction between telmisartan and hydrochlorothiazide in spontaneously hypertensive rats. J. Pharm. Pharmacol..

[44] Asdaq S.M., Inamdar M.N. (2009). The potential for interaction of hydrochlorothiazide with garlic in rats. Chem. Biol. Interact..

[45] Fujimura A., Shiga T., Ohashi K.-i., Ebihara A. (1993). Chronopharmacology of amlodipine in rats. Life Sci..

